# Use of participatory action research approach to develop a self-management resource for persons living with dementia

**DOI:** 10.1177/1471301221997281

**Published:** 2021-02-25

**Authors:** Sherry Dupuis, Carrie McAiney, Lisa Loiselle, Brenda Hounam, Jim Mann, Elaine C Wiersma

**Affiliations:** Department of Recreation and Leisure Studies and Partnerships in Dementia Care Alliance, 8430 University of Waterloo, Ontario, Canada; School of Public Health and Health Systems and Partnerships in Dementia Care Alliance, 8430 University of Waterloo, Ontario, Canada; Murray Alzheimer Research and Education Program, 8430 University of Waterloo, Ontario, Canada; Partner living with dementia of the Murray Alzheimer Research and Education Program, 8430 University of Waterloo, Ontario, Canada; Partner living with dementia of the Murray Alzheimer Research and Education Program, 8430 University of Waterloo, Ontario, Canada; Department of Health Sciences and the Centre for Education and Research on Aging & Health, Lakehead University, Ontario, Canada

**Keywords:** participatory action research, self-management, knowledge translation, dementia

## Abstract

This article describes the use of a participatory action research (PAR) approach to developing a self-management resource for persons living with dementia and care partners. Despite growing evidence that persons with dementia are able to contribute in meaningful ways to decision-making about their care and life preferences, few opportunities exist for them to participate in the design of resources and services meant for them. There is also a need to support the self-management of persons living with dementia with the provision of accurate, high quality, user-friendly information. The *Living Well with Dementia* resource was developed through a partnership with persons with dementia, family members, Alzheimer Society representatives, primary care providers, and researchers. The methods used in the development of this resource are outlined in six steps employed in this process, from establishment of a PAR team to final resource creation. Informed by a whole systems approach, the resource brings together essential components of self-management into a comprehensive system of care and support for living. It empowers users to be active participants in the application of new knowledge to their lives. Better self-management has important implications for access to health care and quality of life for persons with dementia and care partners.

## Introduction

There is much stigma associated with dementia (Mitchell et al., 2013), and efforts to manage this disease tend to focus on managing functional decline and safety risks, with carers assuming control over decision-making in the best interests of the individual with dementia (Burgener et al., 2015; Riley et al., 2014). There is growing evidence that persons with dementia are able to adapt to functional changes, finding ways to remain active, and create meaning in their lives as they live with this disease (Bartlett 2014a, 2014b; Beard, 2004; Genoe & Dupuis, 2012, 2014; Harman & Clare, 2006). Moreover, persons with dementia are interested in contributing to and sharing in decision-making related to disease management (Dupuis et al., 2012a, 2012b), as evidenced by the development of programs empowering them to assume greater control in their lives and develop skills to better self-manage their care (Dupuis & Gillies, 2014; Hickman, et al., 2015; Wiersma et al., 2017). The importance of engaging persons with dementia in program development has been identified by Alzheimer’s Disease International (2019), the World Health Organization (2015), and country-specific policy documents (Canadian Academy of Health Sciences, 2019; NHS, 2017; Public health Agency of Canada, 2019).

Self-management approaches, especially for persons with chronic conditions, have been identified as an opportunity to inform and empower individuals to maintain well-being and quality of life. In fact, since being introduced in the 1960s, the potential of self-management in the context of chronic diseases has been widely recognized (Allegrante et al., 2019). With a focus on giving people knowledge, skills, and tools needed to make decisions about their health and wellness and assist with role and emotional management, self-management approaches have been recommended as an opportunity to improve health outcomes and reduce healthcare costs (Allegrante et al., 2019). Self-management programs have been developed for numerous chronic conditions including arthritis, asthma, diabetes, cardiovascular disease, depression, and HIV and have demonstrated positive outcomes for many, although not all, conditions (e.g., Allegrante et al., 2019; Bosworth et al., 2009; McCauley et al., 2006; Van Grieken et al., 2018; Vas et al., 2017; Warner et al., 2015, 2019). Although the concept of self-management has been defined in a number of different ways, most definitions include three key components: (1) management of the medical issues associated with the disease; (2) maintaining meaning in life and valued roles; and (3) managing the emotional responses associated with the diagnosis (Allegrante et al., 2019; Wiersma et al., 2017).

Given the projected increase in dementia prevalence and growing interest in supporting self-management among persons with dementia, it has never been more imperative to develop effective means for supporting persons living with dementia. This article describes the participatory action research (PAR) process used in the development of a self-management resource designed by and for individuals with dementia as part of a broader system of support. It is our hope that our process might inspire and inform other participatory processes inclusive of people living with dementia or other individuals often excluded from research and knowledge translation initiatives.

## Self-management in dementia

As persons living with dementia have experiences similar to persons living with other chronic conditions related to the management of medical issues and emotional responses to the disease and the challenges of maintaining meaning in life (Mountain, 2006), self-management approaches have the potential to significantly enhance quality of life for persons with dementia and their care partners. There is evidence that early support and information may reduce fears and anxieties related to the diagnosis, help to maintain self-esteem, increase feelings of control, and inspire hope and new possibilities for living well for persons living with dementia (Dupuis & Gillies, 2014; Sørensen et al., 2008); reduce the adverse outcomes associated with the caring role for family members and better prepare them for the future (Boots et al., 2015; Kjallman Alm et al., 2013; Sørensen et al., 2008); and reduce the societal costs of long-term care (Leifer, 2003). Despite the potential value of such programs for persons living with dementia, the concept of self-management in the dementia context has received limited attention.

A key component of self-management is access to information about the disease and the supports needed to live well. In the case of dementia, such information is needed at all phases of the dementia journey, particularly at the point of diagnosis. While persons with dementia, especially in the early phases of the condition, can take on an active part in their treatment and future planning, this can only happen when persons living with dementia and family members have access to adequate information and supports to do so. Yet, much of the information provided tends to be focused at family members; very little is known about the information needs of persons living with dementia, and few relevant resources exist to address these information needs (Alzheimer Society, 2010; Killen et al., 2016).

The lack of attention to self-management in dementia and the information needs specific to persons living with dementia have been attributed to a number of issues including stigma and the predominant misunderstanding that persons with dementia are unable to learn new skills and lack the capacity to contribute to their own care (Mountain, 2006). These perceptions pervade despite research demonstrating the continued abilities of persons with dementia to describe their experiences and participate in decision-making about their care and the increasing direct involvement of persons with dementia in advocacy initiatives and in educating others with dementia (Dupuis et al., 2012a, 2012b; Dupuis & Gillies, 2014; Bartlett, 2014a, 2014b). Thus, there are growing calls for more research on the information needs of people living with dementia (Alzheimer Society, 2010) and for self-management approaches in dementia (Hickman et al., 2015, 2016; Mountain, 2006; Vernooij-Dassen et al., 2005; Vernooij-Dassen & Olde Rikkert, 2004; Wiersma et al., 2017). In fact, understanding and meeting the information needs of persons living with dementia to support self-management have been identified as pressing issues in provincial (Ontario Ministry of Health and Long-Term Care, 2016) and national dementia strategies (Alzheimer Euorpe, 2017) and as an essential component of dementia-friendly communities (Dupuis, 2010).

## Self-management approaches and resources

There is some evidence that educational resources such as “resource kits,” toolkits, and online resources may be an efficient and cost-effective way to provide educational support to persons newly diagnosed with chronic illnesses (Allegrante, 2019; Lorig et al., 2013, 2015) and could have possibilities for supporting other populations experiencing a new diagnosis, such as persons living with dementia. Educational resources have been used effectively to assist physicians in educating their clinical teams (Schwartzberg et al., 2005) and have been evaluated for their use with specific populations including arthritis and fibromyalgia (Lorig et al., 2008), osteoarthritis (Umapathy et al., 2015), and other chronic illnesses (Lorig et al., 2013). However, no research, to the best of our knowledge, has been conducted on the use of similar print and/or internet resources with persons with dementia. This knowledge translation gap demonstrates not only the great need for high quality, reliable, relevant, clearly communicated, person-centered information for older adults and others newly diagnosed with an illness or disability and their families (Harris et al., 2002) but also how clinicians often underestimate the amount of information their patients want or require to adjust to their illness (Kinnersley et al., 2007).

Self-management resources bring together information and resource tools that have been reviewed for readability, relevance and usefulness to the target population, and accuracy of information. Reliable information can help individuals diagnosed with an illness or disability feel empowered and more in control of their situations, enables them to become more knowledgeable about their illness and the care and treatment options, increases awareness of the supports and services available, promotes active involvement in decision-making and making more informed choices, supports higher levels of self-care, relieves anxiety, helps in the management and in coping with their illness, and increases satisfaction with care (Allegrante et al., 2019; Berzins et al., 2009; Boger et al., 2015; Dineen-Griffin et al., 2019).

Although a dementia-specific resource may be a significant opportunity for persons living with dementia to better participate in their own care and decision-making, information alone may be insufficient to impact ability to manage health and enhance quality of life. A criticism of psychological models of self-management is that they often lay sole responsibility for management of disease on individuals without considering the broad systems and programs needed to support individuals in self-care (Northrup, 2002). Instead, it has been recommended that the focus should be on identifying and drawing together the different components essential to self-management into a seamless, comprehensive, and coherent system of care and support (Kennedy & Rogers, 2001; Kennedy et al., 2014; Mountain, 2006; Northrup, 2002). A whole systems approach to self-management would bring together a strong network of support, including family care partners, community resources and programs, and tools to support self-management such as information provision and knowledge, the empowerment of individuals living with chronic illness so they can be actively involved in decision-making and problem-solving, and the design of appropriate and effective care and support services that are flexible, easy to access, and needs-based. Indeed, information tools found to be the most effective and successful are those that are developed with individuals with lived experience (Bombard et al., 2018). But to be effective, self-management requires a strong partnership between persons with chronic illness, their family care partners, and healthcare providers, as well as access to a full system of programs and services to support self-management both at the system level and the individual level.

### Development of a Living Well with Dementia resource using PAR

The focus of this project was to bring together persons living with dementia, family care partners, and a diverse group of professionals and researchers to develop and implement a self-management resource for persons living with dementia and their family care partners. A multi-phased needs assessment and priority setting exercise conducted by the Murray Alzheimer Research and Education Program (MAREP), University of Waterloo, found that there was overwhelming consensus among persons living with dementia, family members, and health professionals regarding the need for relevant and accessible information at the point of a dementia diagnosis. People with dementia and their family members involved in the process described often feeling isolated and confused after getting a diagnosis of dementia and yet did not know where to turn for reliable information. A commitment was made at the end of the priority setting process to work together in creation of an information tool that would be provided to families at diagnosis. It was our hope that supporting self-management for persons living with dementia would mean that individuals would be better able to work with their healthcare team, more prepared to participate in decisions, more aware of available supports, more knowledgeable of treatments, more actively involved in their own care for longer periods of time, have the resources necessary to deal with the emotional aspects of the diagnosis, and ultimately be better able to live well with dementia. A unique aspect of dementia self-management results from the increasingly important role of family care partners as dementia progresses and cognitive function declines. For family members, better self-management may result in less carer stress and easier access to key information to help carers in their roles and be better prepared to plan ahead and support the person living with dementia.

If persons with dementia and their family care partners are to take more of an active role in self-management, then integrated knowledge translation (IKT) is a fundamental prerequisite to their involvement and participation. IKT refers to collaboration between researchers and individuals who use the research (Gagliardi et al., 2016). However, IKT is rarely used in the dementia context, especially where people with dementia are actively included in the process. In fact, traditional approaches to knowledge translation often referred to as “knowledge to action” approaches fail to recognize the important contributions that persons living with illness or disability can make to the knowledge generation and sharing processes, and how knowledge generation/creation can happen simultaneously with knowledge use/transfer referred to as “knowledge in action” (Dupuis & Whyte, 2017). For these reasons, we were drawn to participatory action research (PAR) for this project.

Although people with dementia were rarely included in decision-making in research processes prior to 2000, participatory approaches that actively engage people living with dementia in the research process have been increasing over the past two decades, including projects conducted in Japan (Nomura et al., 2009), Australia (Goeman et al., 2016), Canada (Dupuis et al., 2012a, 2012b; Fortune et al., 2015; Hickman et al., 2015, 2016; Mann & Hung, 2019; McKeown et al., 2015), the UK (Rodgers, 2018), and Belgium (Hendriks et al., 2013). Participatory processes have helped inform guidelines and frameworks and identify innovative approaches and strategies for supporting people with dementia as co-researchers, co-designers (Dupuis et al., 2012a; Hendriks et al., 2013; Phillipson & Hammond, 2018; Wang et al., 2019) and as co-analysts (Clarke et al., 2018), including people living with dementia in later phases of dementia (Smith & Phillipson, 2020). Although some participatory approaches have included people living with dementia in a specific phase of the process (i.e., predesign or evaluative phases), more recent studies have been engaging people living with dementia in co-design in all phases of the process (Wang et al., 2019). This research demonstrates that meaningful inclusion of people living with dementia in research design is not only possible but also beneficial to both people living with dementia and the design of the research (Wang et al., 2019).

There were five common elements of PAR that made it an appealing methodology for our process. People experiencing specific phenomena or working in particular settings are included as co-researchers throughout the process.

This means actively engaging all in meaningful ways in collaborative decision-making; collecting and analyzing data; reflecting, interrogating, and interpreting the information; determining the best ways to share information and in constructing the story to be told; and acting on the information (Dupuis & Whyte, 2017, p. 106; Kemmis et al., 2014)

Understanding what is of practical significance to the people and settings involved and drawing on local knowledge and methods is of utmost importance (Reason & Bradbury, 2008). PAR integrates knowing and acting in the same process, making both knowledge co-creation and action critical dimensions of PAR processes (Dupuis & Whyte, 2017). PAR often has a social justice agenda, with the ultimate goal of personal and social transformation that happens as a result of engaging in PAR processes. In fact, Reason and Bradbury (2008) suggest that “[a]ction research without its liberating and emancipatory dimension is a shadow of its full possibility and will be in danger of being co-opted by the status quo” (p. 5). Finally, PAR is a fluid and emergent process that unfolds over time as new information is gathered, reflected upon, and acted on (Dupuis & Whyte, 2017; Reason & Bradbury, 2008). What follows is a description of the action research cycles and steps undertaken to develop a dementia resource that actively engaged diverse voices, particularly persons living with dementia, in the development and implementation of this resource.

### Establishment of a PAR team

The first step in developing the *Living Well with Dementia* resource was to develop a PAR team. Guided by the principals of PAR and the authentic partnership model developed by Dupuis et al. (2012a), 17 individuals with diverse backgrounds and experiences were brought together to form this team and to oversee the development of the resource. As Dupuis et al. (2012a) outline, an authentic partnership

1. Recognizes how persons with dementia have been silenced, excluded, and oppressed while at the same time recognizes the collective capacity they have to empower themselves and others (Foley, 2001; Freire, 1972, 1976);
2. Seeks to work in partnership with persons with dementia, their families, and others to promote equality and social justice for all persons with dementia (Adams & Clarke, 1999; Foley, 2001; Freire, 1972);
3. Views knowledge as power and education and learning as important vehicles for social change, transformation, and liberation (Findsen, 2007; Freire, 1972, 1976);
4. Challenges the supremacy of higher order or expert knowledge and instrumental rationality (Habermas, 1984) by acknowledging, valuing, and incorporating the lived or experiential knowledge of our partners (Thomas, 1982); and
5. Incorporates a systematic process of critical reflection and dialog in community with others as all partners work collectively toward the realization of new possibilities (Freire, 1972; Habermas, 1984). (p. 432)

Thus, our PAR process brought together persons with dementia, family care partners, Alzheimer Society representatives and educators, First Link Coordinators (i.e., professionals who work to link individuals and families experiencing dementia to community supports and services; see McAiney et al., 2008, 2012), primary care physicians, and other health professionals working with persons with dementia such as people working in pharmacy and an interdisciplinary team of researchers from diverse disciplines/fields. In recruiting PAR team members, we started by identifying and inviting individuals from a range of organizations and backgrounds who had previously engaged in PAR processes with us and had demonstrated a willingness and commitment to participatory processes. Consistent with authentic partnerships (Dupuis et al., 2012a), we used early meetings to reflect on who was included and who might be missing and should be invited to participate. Throughout our reflective process, when it was determined that expertise might be missing from the team and needed, we searched out potential new members and invited them to join.

**Figure 1.**
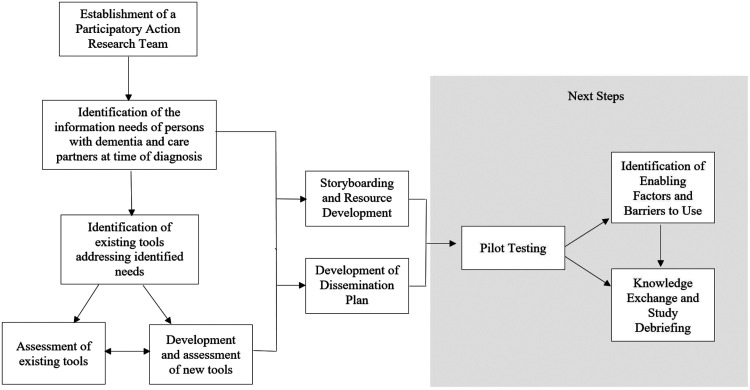
Summary of steps involved in developing the *Living Well with Dementia* resource.

In early meetings and/or separate conversations with team members, we also explored the expertise, strengths, and talents they brought to the project and how they preferred to be involved (Dupuis et al., 2012a). Then, together, and in regular monthly meetings, the team worked collaboratively to identify and carry out the subsequent PAR cycles and steps required to develop the resource; these steps are summarized in Figure 1. Our process was documented using detailed minutes from all team meetings and process activities, which were shared regularly with all team members. We also used our meetings to critically reflect on our process and determine collectively how to move forward.

In consultations with the Office of Research Ethics at one of the PIs universities and consistent with practices used in PAR processes at that university, we prepared and received ethics clearance for this project at the outset of the project (ORE 15805) and for all phases of the project where data were being collected and analyzed through the submission of modifications to the original application. Full ethics clearance was also obtained for the pilot testing of the resource at both PIs universities (ORE# 17953, REB# 12-030). Our ethics process was informed by Dewing’s (2007) work with persons living with dementia and Welsby and Horsfall’s (2011) research with women with intellectual abilities, both of whom recommend the use of options and flexibility in securing consent and/or assent and the continuous use of process consent procedures to ensure all partners and participants are reminded of the project purpose and have opportunities to make informed decisions about their continued involvement in each phase of the project.

### Identification of information needs

The PAR team determined that the first step in the process was to identify the information needs of persons living with dementia and their care partners at the time of diagnosis. In order to do this, a variety of needs assessment methods were chosen including two focus groups with participants (*N* = 8 participants) in early-stage support groups facilitated by a person with dementia and a researcher from our team; face-to-face or telephone interviews with persons with dementia, family members, and professionals (*N* = 14); and open-ended questionnaires completed by persons with dementia (*N* = 21) and family members (*N* = 17). Questions were posed about what types of information were needed at the time of diagnosis, what information was most helpful, preferences for accessing information, and the experience of interacting with the healthcare professionals when diagnosed. These methods provided an opportunity to explore and identify the types of information people with dementia felt they needed but had little access to.

Working together, the team then reviewed and analyzed the data generated from this process and used the data to develop a second questionnaire called “Identifying Your Information Needs and Preferences.” A draft version of the new questionnaire was shared with 18 persons living with dementia, five family care partners, and one professional to ensure the information was understandable and the questionnaire was easy to complete. On a five-point Likert scale from “very important” to “not important,” participants were asked to indicate how important each type of information was at the time of diagnosis. The questionnaire identified specific types of information in different areas including the diagnosis process, dementia, healthcare systems and teams, treatments and medication, emotional needs, communication, community supports and services, quality of life, planning ahead, safety, and care options. Participants were also asked to indicate how they prefer to receive information, where they prefer to access information, from whom they prefer to receive information, and when they would prefer to receive information. This second questionnaire was made available in both hard copy and online versions and distributed in a range of ways including through the MAREP contact database, the Alzheimer Society of Ontario, Alzheimer Society of Canada, and the Dementia Advocacy and Support Network International (now known as Dementia Alliance International). Hard copies of the questionnaire were also distributed at two *A Changing Melody* forums designed specifically by and for people living with dementia and their care partners. A total of 115 persons with dementia and family care partners completed the questionnaire.

Information generated through these efforts was consistent with previous research on chronic illnesses indicating a common core set of information needs of older persons newly diagnosed (Abdi et al., 2019; Grobosch et al., 2018; Harris et al., 2002; McGilton et al., 2018). Across all of these sources of information, participants identified a range of information needs that fit into four broad themes related to *health care* (e.g., information about illnesses causing dementia, how to recognize symptoms and how to act upon symptoms, how to work with healthcare teams, and medical and nonmedical treatments); *care and support* (e.g., the availability of social and other support services and how to access those services, safety considerations, and care options including long-term care placement); *living well* (e.g., how to transform and live with the emotional reactions of the diagnosis such as grief, loss, and anger, how to enhance communication, and how to maintain holistic wellness and quality of life in order to live well with the diagnosis); and *planning ahead* (e.g., how to manage activities and strategies for planning ahead including dealing with financial consequences of the diagnosis, designating powers of attorney, and end of life care). These themes are similar to those found in a recent study by Soong et al. (2020). Given the differences in terms of availability of support services and processes for accessing those services across Canada, the PAR team had a number of conversations at this stage to determine how best to discuss information related to the theme of care and support. It was decided that it would be extremely challenging to outline processes for all jurisdictions across the country and that, where possible, we would provide generic information about support services as well as more specific information relevant to Ontario citizens where many on our team were located.

### Identification of existing self-management resources

As the information needs of people living with dementia and their care partners were being explored, the team also acknowledged that some good resources may already be in existence but that it was often difficult to locate these resources. We determined that it was important to build on existing resources where possible and not “reinvent the wheel.” With direction from the PAR team, MAREP staff members began conducting a comprehensive environmental scan in order to identify all existing resources at the time being used to provide information to persons with dementia and their care partners. This task was done by conducting a thorough internet search and interviewing health professionals and Alzheimer Society staff across the country about the tools and resources they were currently using. When new tools or resources were identified, a request was made to send a copy to MAREP. Over 500 individual resources were identified.

### Assessment of existing tools and resources

Coulter (1998) highlighted the important role that information materials can play in supporting self-management but also stressed: “If patients are to be active participants in decisions about their care, the information they are given must accord with available evidence and be presented in a form that is acceptable and useful” (p. 225). Unfortunately, few resources meet the standards for high quality, usefulness, and relevance because in most situations, resources are developed without people with lived experience being actively involved in their development. Our team determined that an evaluation process was needed to assess the accuracy, readability, relevance, and usefulness of the existing resources to persons living with dementia and that persons living with dementia must be included in that process. A subcommittee was created to develop assessment tools/workbooks that could be used for this purpose, manage the evaluation of existing resources, and ensure that assessors were trained to use the assessment tools/workbooks.

Consistent with authentic partnerships (Dupuis et al., 2012a), at the beginning of our process, we explored with team members what talents, skills, strengths, and expertise they felt they brought to the project, and as we progressed, we continued to check in to determine how diverse team members wanted to be included. At this phase, one of our team members with dementia identified an interest and willingness to participate on the subcommittee and also in the assessment process; another team member with dementia preferred to be included only in the assessment process. We also were able to engage students who expressed an interest in engaging in our research process as members of the subcommittee and/or assessors. This resulted in a subcommittee made up of one educator, three researchers, one person with dementia, and three students (two undergraduates and one doctoral candidate).

Two assessment tools/workbooks were developed for this purpose: one to be used by assessors who did not have dementia and one to be used by persons with dementia. The assessment tools/workbooks were based on existing valid and reliable measures used to assess these factors, including the DISCERN and SAM tools (Charnock & Shepperd, 2004; Charnock et al., 1999; Doak et al., 1994) as well as suitability for individuals with low literacy skills (Doak et al., 1996). Readability was assessed using the online SMOG tool (https://www.readabilityformulas.com/smog-readability-formula.php). Six assessors, including two persons with dementia, two undergraduate students, one doctoral candidate, and one master’s level student, were first trained and then collaborated in the assessment of all 500+ existing resources using the assessment tools and workbooks created by the subcommittee. Once the assessment process was completed, the team had a better sense of the information areas where strong resources and tools already existed that could be incorporated into our resource, and a list of gaps in the tools available to address the information needs of persons with dementia was created (see Table 1).

**Table 1.** Identified Information Need Requiring Content to be Developed.

Category	Identified needs
Health care	How dementia affects coexisting medical conditions
	The purpose of certain tests
	The length of time testing will take
	How to prepare for tests (before and after)
	What test results mean
	How to get referred to a specialist
	Available specialists in your area
	What to expect at a specialist appointment
	What you can do while waiting to get a specialist appointment (e.g., tips on how you can help yourself protect your memory)
Care and support	How different health professionals can help
	Which services are appropriate at different stages of the dementia journey
	Cost of service/wait time
	How to navigate the healthcare system
	Safety assessments
Living well	Specific strategies for dealing with anger
	Strategies for dealing with other responsibilities (e.g., caring for parents or children)
Planning ahead	When/how to give up responsibilities (e.g., driving, cooking, and being alone)
	How dementia may affect insurance policies
	Emergency care plan
	The long-term care home placement process (e.g., wait times)
	Cost of long-term care
	What to expect on moving day
	Transition periods

### Development of new resources/information to address gaps

Within the PAR team, small working groups were developed to gather information needed to create the resources (or content) for those areas where no quality and/or useful resources existed. This information was gathered from comprehensive literature searches and individual interviews with expert informants. As an example, one of the information areas identified by persons with dementia in which no high quality resource existed was what to expect at a specialist appointment. Family physicians and specialists working in memory clinics were consulted to determine, with our partners with dementia, the information most relevant to include. We then worked with our partners with dementia and family members to identify helpful ways to present this information so that it was accessible and relevant to persons with dementia and their care partners. Draft versions of the content were reviewed by the PAR team, and once new content was finalized, the same procedures used to analyze the existing resources were used to assess the new content, including an assessment for relevance, usefulness, readability, and accuracy of information.

### Creation of the Living Well with Dementia online resource

In addition to determining information needs of persons with dementia early in our process, participants involved in the needs assessment were also asked how and where they preferred to receive information and when in the process they preferred it. The majority of persons with dementia (84%) indicated they preferred to receive information they could read on their own. They preferred to receive information either in a support setting (79%) or in their home (71%). In addition, the majority of persons living with dementia (66%) indicated they preferred having information staggered over time, available in a way that would provide them with control in choosing what they looked at when. This approach was important for reducing feeling overwhelmed, especially when too much information was provided at once. However, 36% of participants indicated a preference to receive all information at once at the time they were diagnosed. Using this information, the team then explored all possible options for providing the information we had gathered and created to persons with dementia and their care partners. After much discussion and critical reflection, the PAR team made the decision to create a web-based resource that would enable individual users to access information in a way each is most comfortable, at a time relevant to them; allow easy access to individuals working in support settings to access the information and print copies for individuals who prefer written materials; enable updates of the resources to be made quickly and easily, thereby allowing the information to remain current and accurate; and reduce the high costs associated with printing large volumes of information.

Written material has become one important venue for providing supplementary information to persons living with illness or disability (Allegrante et al., 2019). Written materials allow the learning process to continue after consultation, can be used to reinforce information provided verbally, as well as provide additional information that cannot be relayed during consultations, and provide opportunities for individuals to absorb information at their own pace (Coulter, 1998). However, written materials can often be difficult to get into the hands of persons newly diagnosed, leaving many without the information they need for effective self-management. Internet-based resources are increasingly becoming a primary source of information and advice (Allegrante et al., 2019), including with persons living with dementia and their care partners (Soong et al., 2020) and older adults (Flynn et al., 2006: Medlock et al., 2015). Advantages of sharing information via the internet include increased accessibility, interactivity, information tailoring, privacy and anonymity, and providing a more graphically and aesthetically engaging experience (Allegrante et al., 2019). Moreover, internet-accessed health-related information can have a significant impact on outcomes for persons experiencing illness, enabling them to engage more directly with and have greater responsibility in their healthcare decisions, and increase awareness about the supports and resources available to them (Allegrante et al., 2019; Flynn et al., 2006).

In order to design a successful website, it was necessary to do some pre-planning, including the development of a website storyboard. Storyboarding involves determining the information important to be shared and creating and diagramming a site structure that best fits user needs (Techwalla). As part of the website development process, an in-person storyboarding meeting was held with the PAR team. Over the course of the daylong event, members of the PAR team discussed the purpose of the website, the target audience(s), and accessibility features that needed to be incorporated into the site (e.g., inclusion of subtitled videos, print, and +/− zoom function). More detailed conversations occurred to determine preferences for organizing the website, graphics, color scheme, navigation, and content. Our partners with dementia were actively involved in choosing the layout and design features that best supported navigation of the site; the colors used on the site so that they were warm and inviting; the development of a butterfly logo for the site that they felt represented hope; options to increase font size; the ability to print each page if desired; and the photos chosen for the site. Our partners also felt that it would be important to include videos as an alternative way of sharing information and helping people living with dementia navigate the site and helped us in developing new videos and identifying existing videos they found useful.

Using decisions made during the storyboarding process, the final step in the development phase involved creating the website, posting the content onto the new site, and doing an initial testing of the usability and accessibility of the draft site with our partners living with dementia and care partners. This process provided us with an important opportunity to review the entire site through the eyes of people it was designed for, identify issues that might exist relevant to navigating the site and understanding content provided on the site, and make final tweaks to the site before launching it and embarking on a broader pilot study. The resulting resources developed for this initiative can be accessed at https://uwaterloo.ca/living-well-with-dementia//. All resources were also translated into French.

### Developing a plan for disseminating the Living Well with Dementia web-based resource

One of the concerns raised by our partners with dementia was around how we were going to ensure that people with dementia were made aware of the resource at the time of diagnosis. It was important that this resource be embedded within the larger community healthcare system so that people could be made aware of it and supported in using it when needed. We worked with our team members living with dementia to identify the places/people they are most likely to access at diagnosis or shortly after. We also explored with the professionals on our team what they needed in order to share and use the resource. Based on this information, the PAR team felt that it was critical that family physicians and memory clinics, local pharmacists, First Link Coordinators (where they exist), and Alzheimer Society staff be made aware of the resource and be able to provide persons living with dementia and their family care partners with something they could take home with them that would direct them to the resource. To this end, we worked with our PAR team to create *Living with Dementia* postcards that introduced the resource and provided information on how to access it. A “Frequently Called Numbers” magnet was also created that provided spaces for people living with dementia to put the telephone numbers for their physicians, the local hospital, their pharmacist, the local Alzheimer Society, and other important numbers. The magnet also included the information for accessing the *Living Well with Dementia* resource. In a large dissemination initiative, these postcards and magnets, in both English and French (Canada’s two official languages), were distributed to the individuals/organizations identified as key resources by people with dementia. A USB/memory key wristband was also created with more detailed information about the resource. When people with dementia used the USB, they had the option of reading the information or listening to an audio file of the information. People with dementia were able to access the resource directly using the link provided on the USB. These resources were distributed at several conferences, meetings, and workshops. Finally, members of the team also arranged to attend conferences of key professional groups including a meeting with the First Link Coordinators and with Alzheimer Society educators to make them aware of the new resource and discuss with them what they needed to better refer the resource to the people they were working with.

## Final thoughts on our process

Increasingly, research is showing that persons living with dementia can be active participants in their own care and decisions about care and preferences for living (Dupuis et al., 2012a, 2012b; Wiersma et al., 2017). Despite often being excluded from research and knowledge translation initiatives, our process not only demonstrates that people with dementia can participate in active and meaningful ways as co-researchers in PAR processes but also how inclusion of persons with dementia ensures the relevancy of the decisions made and actions taken to persons living the experience. People with dementia were included in all aspects and phases of this process; without them, this resource would have been something very different and likely not as useful to them. Our approach is consistent with conceptual frameworks for self-management that emphasize the importance of providing resources and making linkages for people with dementia and their families but also support the empowerment of persons with dementia to be agents of social change in the social structures within the context of their unique lives (Bartlett, 2014a, 2014b; Dupuis & Gillies, 2014; Wiersma et al., 2017). The next phase in our PAR process was the piloting of the resource with people with dementia and their family members, and the team is currently working on an article that describes this process and the results of the pilot.

An additional outcome of this process was the identification of the need for a self-management education program that would be developed and facilitated by people living with dementia. Led by Dr. Elaine Wiersma, members of our team were successful in obtaining funding to develop the *Taking Control of Our Lives* program, a self-management face-to-face education program designed by and for persons living with dementia using PAR. The program explores various topics related to dementia and encourages participants to increase understanding, develop skills, and feel more confident in making decisions about their own care and living (Hickman et al., 2016; Wiersma et al., 2017). Innovations like the *Living Well with Dementia* resource and *Taking Control of Our Lives* program, and using PAR processes as a means of building strong partnerships between individuals and organizations in communities central to supporting people living with dementia, bring together different components essential to self-management in order to create a more comprehensive and coordinated system of support for persons with dementia and their care partners (Mountain, 2006; Northrup, 2002). Including diverse stakeholders in our process served as an important step in building and strengthening networks for our partners with dementia and their care partners.

## References

[bibr1-1471301221997281] Abdi S. Spann A. Borilovic J. de Witte L. Hawley M. (2019). Understanding the care and support needs of older people: A scoping review and categorisation using the WHO international classification of functioning, disability and health framework (ICF). BMC Geriatrics, 19, 195. doi:10.1186/s12877-019-1189-9 31331279 PMC6647108

[bibr2-1471301221997281] Adams T. Clarke C. L. (1999). Dementia care: Developing partnerships in practice. Baillie`re Tindall.

[bibr3-1471301221997281] Alzheimer Europe (2017). National Dementia Strategies: A snapshot of the status of national dementia strategies around Europe. Luxembourg: Alzheimer Europe. http://www.alzheimer-europe.org/Policy-in-Practice2/National-Dementia-Strategies

[bibr4-1471301221997281] Allegrante J. Wells M. Peterson J. (2019). Interventions to support behavioural self-management of chronic diseases. Annual Reviews Public Health, 40, 127-146. doi:10.1146/annurev-publhealth-040218-044008 PMC6684026 30601717

[bibr5-1471301221997281] Alzheimer Society (2010). Information need of people with dementia and carers. Alzheimer Society.

[bibr6-1471301221997281] Alzheimer's Society of Canada (2016). Prevalence and monetary costs of dementia in Canada. Alzheimer's Society of Canada. https://alzheimer.ca/sites/default/files/files/national/statistics/prevalenceandcostsofdementia_en.pdf

[bibr7-1471301221997281] Alzheimer’s Disease International (2019). World Alzheimer report 2019: Attitudes to dementia. Alzheimer’s Disease International. https://www.alzint.org/u/WorldAlzheimerReport2019.pdf

[bibr8-1471301221997281] Hickman K. Wiersma E. C. Harvey D. (2015). Taking control of our lives: Developing a self-management education program for people living with dementia through meaningful engagement of people living with dementia. Alzheimer's & Dementia, 11(Suppl 7), pp. 191-192. doi:10.1016/j.jalz.2015.07.173

[bibr9-1471301221997281] Hickman K. Wiersma E. C. Loiselle L. (2016). Empowering people with early stage dementia to live well through a Dialogue Education™ approach. Alzheimer's & Dementia, 12(Suppl 7), pp. 301-302. doi:10.1016/j.jalz.2016.06.545

[bibr10-1471301221997281] Bartlett R (2014a). The emergent modes of dementia activism. Ageing and Society, 34(4), 623-644. doi:10.1017/S0144686X12001158

[bibr11-1471301221997281] Bartlett R . (2014b). Citizenship in action: the lived experiences of citizens with dementia who campaign for social change. Disability & Society, 29(8), 1291-1304. doi:10.1080/09687599.2014.924905

[bibr12-1471301221997281] Beard R. (2004). In their voices: Identity preservation and experience of Alzheimer's disease. Journal of Aging Studies, 18, 145-428. doi:10.1016/j.jaging.2004.06.005

[bibr13-1471301221997281] Berzins K. Reilly S. Abell J. Hughes J. Challis D. (2009). UK self-care support initiatives for older patients with long-term conditions: A review. Chronic Illness, 5(1), 56-72. doi:10.1177/1742395309102886 19276226

[bibr14-1471301221997281] Boger E. Ellis J. Latter S. Foster C. Kennedy A. Jones F. Fenerty V. Kellar I. Demain S. (2015). Self-Management and self-management support outcomes: A systematic review and mixed research synthesis of stakeholder views. Plos One, 10(7), Article e0130990. doi:10.1371/journal.pone.01309902469-2475 26162086 PMC4498685

[bibr15-1471301221997281] Bombard Y. Baker G. R. Orlando E. Fancott C. Bhatia P. Casalino S. Onate K. Denis J. Pomey M. (2018). Engaging patients to improve quality of care: A systematic review. Implementation Science, 13, 98. doi:10.1186/s13012-018-0784-z 30045735 PMC6060529

[bibr16-1471301221997281] Boots L. M. Wolfs C. A. Verhey F. R. Kempen G. R. Vugt M. E. (2015). Qualitative study on needs and wishes of early-stage dementia caregivers: the paradox between needing and accepting help. International Psychogeriatrics, 27(6), 927-936. doi:10.1017/S1041610214002804 25566686

[bibr17-1471301221997281] Bosworth H. B. Olsen M. K. Dudley T. Orr M. Goldstein M. K. Datta S. K. Mccant F. Gentry P. Simel D. L. Oddone E. Z. (2009). Patient education and provider decision support to control blood pressure in primary care: A cluster randomized trial. American Heart Journal, 157(19), 450-456. doi:10.1016/j.ahj.2008.11.003 19249414

[bibr18-1471301221997281] Burgener S. C. Buckwalter K. Perkhounkova Y. Liu M. F. (2015). The effects of perceived stigma on quality of life outcomes in persons with early stage dementia: Longitudinal findings Part 2. Dementia, 14(5), 609-632. doi:10.1177/1471301213504202 24339117

[bibr19-1471301221997281] Canadian Academy of Health Sciences . (2019). Improving the quality of life and care of persons living with dementia and their caregivers. The Expert Panel on Dementia Care in Canada, CAHS. https://cahs-acss.ca/wp-content/uploads/2019/04/REPORT.pdf

[bibr20-1471301221997281] Charnock D. Sheppard S. Needham G. Gann R. (1999). DISCERN: An instrument for judging the quality of written consumer health information on treatment choices. Journal of Epidemiology and Community Health, 53(2), 105-111. doi:10.1136/jech.53.2.105 10396471 PMC1756830

[bibr21-1471301221997281] Charnock D. Shepperd S. (2004). Learning to DISCERN online: Applying an appraisal tool to health websites in a workshop setting. Health Education Research, 19, 440-446. doi:10.1093/her/cyg046 15155597

[bibr22-1471301221997281] Clarke C. L. Wilkinson H. Watson J. Wilcockson J. Kinnaird L. Williamson T. (2018). A seat around the table: Participatory sata analysis with people living with dementia. Qualitative Health Research, 28(9), 1421-1433. doi:10.1177/1049732318774768 29766747

[bibr23-1471301221997281] Coulter A. (1998). Evidence based patient information. Is important, so there needs to be a national strategy to ensure it. British Medical Journal, 317(7153), 225-226. doi:10.1136/bmj.317.7153.225 9677206 PMC1113581

[bibr24-1471301221997281] Dewing J. (2007). Participatory research: A method for process consent with persons who have dementia. Dementia, 6(1), 11-25. doi:10.1177/1471301207075625

[bibr25-1471301221997281] Dineen-Griffin S. Garcia-Cardenas V. Williams K. Benrimoj S. (2019). Helping patients help themselves: A systematic review of self-management support strategies in primary health care practice. PLoS One, 14(8), Article e0220116. doi:10.1371/journal.pone.0220116 31369582 PMC6675068

[bibr26-1471301221997281] Doak L. G. Doak C. C. Miller K. Wilder L. (1994). Suitability assessment of materials (SAM). American Public Health Association Annual Meeting.

[bibr27-1471301221997281] Doak L. G. Doak C. C. Root J. H. (1996). Teaching patients with low literacy skills*.* Lippincott-Raven. doi:10.1097/00000446-199612000-00022

[bibr28-1471301221997281] Dupuis S. L. (2010). A planning framework for improving the lives of persons with Alzheimer’s disease and related dementias and their families: Implications for social policy, leisure policy and practice. In Mair H. Arai S. M. Reid D. G. (Eds.), Decentring work: Critical perspectives on leisure, social policy and human development, (pp. 91-117). Calgary University Press.

[bibr29-1471301221997281] Dupuis S. L. Gillies J. (2014). Learning as a vehicle for individual and social transformation: Re-thinking leisure education. Therapeutic Recreation Journal, 48(2), 113-134. https://js-sagamorepub-com.proxy.lib.uwaterloo.ca/trj/article/view/5191/4256

[bibr30-1471301221997281] Dupuis S. L. Gillies J. Carson J. Whyte C. Genoe R. Loiselle L. Sadler L. (2012a). Moving beyond ‘patient’ and ‘client’ approaches: Mobilising authentic partnerships in dementia care. Dementia: The International Journal of Social Research and Practice, 11(4), 428-450. 10.1177/1471301211421063

[bibr31-1471301221997281] Dupuis S.L. Whyte C. Carson J. Genoe R. Meschino L. Sadler L. (2012b). Just dance with me: An authentic partnership approach in understanding leisure in the dementia context. Special issue on Leisure, Health and Disability of World Leisure Journal, 54(3), 240-254. doi:10.1080/04419057.2012.702454

[bibr32-1471301221997281] Dupuis S. L. Whyte C. (2017). Participatory approaches to research with marginalized individuals and groups: Ensuring relevancy. In Stumbo N.J. Wolfe B. Pegg S. (Eds) Professional issues in therapeutic recreation (3rd ed). (pp. 101-112). Sagamore Publishing.

[bibr33-1471301221997281] Findsen B. (2007). Freirean philosophy and pedagogy in the adult education context: The case of older adults’ learning. Studies in Philosophy and Education, 26, 545-559. doi:10.1007/s11217-007-9063-1

[bibr34-1471301221997281] Flynn K. Smith M. Freese J. (2006). When do older adults turn to the internet for health information? Findings from the Wisconsin Longitudinal Study. Journal of General Internal Medicine, 21(12), 1295-1301. doi:10.1111/j.1525-1497.2006.00622.x 16995892 PMC1924748

[bibr35-1471301221997281] Foley G. (2001). Radical adult education and learning. International Journal of Lifelong Education, 20, 71–88. doi:10.1080/02601370010008264

[bibr36-1471301221997281] Fortune D. McKeown J. Dupuis S.L. deWitt L. (2015). “It was like reading a detective novel”: Using PAR to work together for culture change. Journal of Aging Studies, 34, 38-47. doi:10.1016/j.jaging.2015.04.002 26162724

[bibr37-1471301221997281] Freire P (1972). Pedagogy of the oppressed. Penguin.

[bibr38-1471301221997281] Freire P (1976). Education: The practice of freedom. Penguin.

[bibr39-1471301221997281] Gagliardi A. R. Berta W. Kothari A. Boyko J. Urquhart R. , (2016). Integrated knowledge translation (IKT) in health care: A scoping review. Implementation Science, 11(38), 1-12. doi:10.1080/14927713.2011.649111 26988000 PMC4797171

[bibr40-1471301221997281] Genoe R. Dupuis S. L. (2012). “I’m just like I always was”” A phenomenological exploration of leisure, identity and dementia. Leisure/Loisir, 35(4), 423-452.

[bibr90-1471301221997281] Genoe R. Dupuis S. L. (2014). “Doing the best I can with what I’ve got”: The role of leisure within the dementia context. Dementia: The International Journal of Social Research and Practice, 13(1), 33-58. 10.1177/1471301212447028 24381038

[bibr41-1471301221997281] Goeman D. King J. Koch S. (2016). Development of a model of dementia support and pathway for culturally and linguistically diverse communities using co-creation and participatory action research. BMJ Open, 6(12), Article e013064. doi:10.1136/bmjopen-2016-013064 PMC5168626 27927662

[bibr42-1471301221997281] Grobosch S. Kuske S. Linnenkamp U. Ernstmann N. Stephan A. Genz J. Begun A. Haastert B. Szendroedi J. Müssig K. Burkart V. Roden M. Icks A. Al-Hasani H. Buyken A. Eckel J. Geerling G. Herder C. Kotzka J., … Ziegler D. (2018). What information needs do people with recently diagnosed diabetes mellitus have and what are the associated factors? A cross-sectional study in Germany. BMJ Open, 8, Article e017895. doi:10.1136/bmjopen-2017-017895 PMC6252653 30385437

[bibr43-1471301221997281] Habermas J. (1984). The theory of communicative action: Reason and the rationalization of society. Beacon Press.

[bibr44-1471301221997281] Harman G. Clare L. (2006). Illness representations and lived experience in early-stage dementia. Qualitative Health Research, 16(4), 484-502. doi:10.1177/1049732306286851 16513992

[bibr45-1471301221997281] Harris M. Bayer A. Tadd W. (2002). Addressing the information needs of older patients. Reviews in Clinical Gerontology, 12(1), 5-11. doi:10.1017/S0959259802012121

[bibr46-1471301221997281] Hendriks N. Truyen F. Duval E. (2013). Designing with dementia: Guidelines for participatory design together with persons with dementia. In Kotzé P. Marsden G. Lindgaard G. Wesson J. Winckler M. (Eds.) Human-Computer Interaction – INTERACT 2013. INTERACT 2013. Lecture Notes in Computer Science, Vol. 8117. Springer. doi:10.1007/978-3-642-40483-2_

[bibr47-1471301221997281] Kemmis S. McTaggart R. Nixon R. (2014). The action research planner: Doing critical participatory action research. Springer.

[bibr48-1471301221997281] Kennedy A. Rogers A. (2001). Improving self-management skills: A whole systems approach. British Journal of Nursing, 10(11), 734-737. doi:10.12968/bjon.2001.10.11.10435 12048491

[bibr49-1471301221997281] Kennedy A. Rogers A. Chew-Graham C. Blakeman T. Bowen R. Gardner C. Lee V. Morris R. Protheroe J. (2014). Implementation of a self-management support approach (WISE) across a health system: A process evaluation explaining what did and did not work for organisations, clinicians and patients. Implementation Science, 9, 129. doi:10.1186/s13012-014-0129-5 25331942 PMC4210530

[bibr50-1471301221997281] Killen A. Flynn C. De Brún A. O’Brien N. O’Brien J. Thomas A. J. McKeith I. Taylor J-P. (2016). Support and information needs following a diagnosis of dementia with Lewy bodies. International Psychogeriatrics, 28(3), 495–501. doi:10.1017/S1041610215001362 26328546

[bibr51-1471301221997281] Kinnersley P. Edwards A. Hood K. Cadbury N. Ryan R. Prout H. Owen D. MacBeth F. Butow P. Butler C. (2007). Interventions before consultations for helping patients address their information needs. Cochrane Database of Systematic Reviews, 3, CD004565. doi:10.1002/14651858.CD004565.pub2 PMC9036848 17636767

[bibr52-1471301221997281] Kjallman Alm A. Hellzen O. Norbergh K. G. (2013). Experiences of long term ongoing structured support in early stage of dementia - a case study. International Journal of Older People Nursing, 9, 289-297. doi:10.1111/opn.12034 23758956

[bibr53-1471301221997281] Leifer B. P. (2003). Early diagnosis of Alzheimer's disease: Clinical and economic benefits. Journal of the American Geriatric Society, 51(Supp 5), S281-S288. doi:10.1046/j.1532-5415.5153.x 12801384

[bibr54-1471301221997281] Lorig K. Ritter P. Laurent D. Plant K. (2008). The internet-based arthritis self-management program: A one-year randomized trial for patients with arthritis or fibromyalgia. Arthritis and Rheumatism, 59, 1009-1017. doi:10.1002/art.23817 18576310

[bibr55-1471301221997281] Lorig K. Ritter P. Moreland C. Laurent D. (2015). Can a box of mailed materials achieve the triple aims of health care? The mailed chronic disease self-management tool kit study. Health Promotion Practice, 16(5), 765-774. 25690615 10.1177/1524839915571633

[bibr56-1471301221997281] Lorig K. Ritter P. Plant K. Laurent D. Kelly P. Rowe S. (2013). The South Australia health chronic disease self-management internet trial. Health Education & Behaviour, 40(1), 67-77. doi:10.1177/1090198112436969 22491008

[bibr57-1471301221997281] Mann J. Hung L. (2019). Co-research with people living with dementia for change. Action Research, 17(4), 573-590. doi:10.1177/1476750318787005

[bibr92-1471301221997281] McAiney C.A. Harvey D. Schulz M. E. (2008). First Link: Strengthening primary care partnerships for dementia support. Canadian Journal of Community Mental Health, 27(2), 117-127.

[bibr94-1471301221997281] McAiney C.A. Hillier L.M. Stolee P. Harvey D. Michael J. (2012). Throwing a Life-Line: The Role of First Link^TM^ in Enhancing Supports for Persons with Dementia and their Care Partners. Neurodegenerative Disease Management, 2(6), 623-638.

[bibr58-1471301221997281] McCauley K. M. Bixby M. B. Naylor M. D. (2006). Advanced practice nurse strategies to improve outcomes and reduce cost in elders with heart failure. Disease Management, 9(5), 302-310. doi:10.1089/dis.2006.9.302 17044764

[bibr59-1471301221997281] McGilton K. Vellani S. Yeung L. Chishtie J. Commisso E. Ploeg J. Andrew M. Ayala A. Gray M. Morgan D. Chow A. Parrott E. Stephens D. Hale L. Keatings M. Walker J. Wodchis W. Dube V. McElhaney J. Puts M. (2018). Identifying and understanding the health and social care needs of older adults with multiple chronic conditions and their caregivers: A scoping review. BMC Geriatrics, 18(1), 231. doi:10.1186/s12877-018-0925-x 30285641 PMC6167839

[bibr60-1471301221997281] McKeown J. Fortune D. Dupuis S.L. (2015). “It’s like stepping into another world”: Exploring the possibilities of using PAR appreciatively to guide culture change. Action Research, 14(3), 1-17.

[bibr61-1471301221997281] Medlock S. Eslami S. Askari M. Arts D. L. Sent D. De Rooij S. E. Abu-Hanna A. (2015). Health information-seeking behavior of seniors who use the Internet: A survey. Journal of Medical Internet Research, 17(1), Article e10. doi:10.2196/jmir.3749 25574815 PMC4296102

[bibr62-1471301221997281] Mitchell G. Dupuis S.L. Kontos P. (2013). Dementia discourse: From imposed suffering to knowing other-wise. Journal of Applied Hermeneutics. doi:10.11575/jah.v0i2.53220

[bibr63-1471301221997281] Mountain G. (2006). Self-management for people with early dementia: An exploration of concepts and supporting evidence. Dementia, 5, 429-446. doi:10.1177/1471301206067117

[bibr64-1471301221997281] NHS (2017). Dementia assessment and improvement framework. https://improvement.nhs.uk/documents/1857/Improving_dementia_care_FINAL_v5_111017.pdf

[bibr65-1471301221997281] Nomura M. Makimoto K. Kato M. Shiba T. Matsuura C. Shigenobu K. Ishikawa T. Matsumoto N. Ikeda M. (2009). Empowering older people with early dementia and family caregivers: A participatory action research study. International Journal of Nursing Studies, 46(4), 431-441. 17983619 10.1016/j.ijnurstu.2007.09.009

[bibr66-1471301221997281] Northrup D. T. (2002). Self-care: Re-examining the myth. In Young L. E. Hayes V. (Eds.), Transforming health promotion practice: Concepts, issues, and applications*.* FA Davis Company.

[bibr67-1471301221997281] Ontario Ministry of health and long-term care (2016). Developing Ontario’s dementia Strategy: A discussion paper. Queen’s Printer for Ontario.

[bibr68-1471301221997281] Phillipson L. Hammond A. (2018). More than talking: A scoping review of innovative approaches to qualitative research involving people with dementia. International Journal of Qualitative Methods, 17(1), 1-13. https://doi.org/10.1177%2F1609406918782784

[bibr69-1471301221997281] Public Health Agency of Canada (2019). A dementia Strategy for Canada: Together we aspire. https://www.canada.ca/content/dam/phac-aspc/images/services/publications/diseases-conditions/dementia-strategy/National%20Dementia%20Strategy_ENG.pdf

[bibr70-1471301221997281] Reason P. Bradbury H. (2008). The SAGE handbook of action research: Participative inquiry and practice. 2nd ed. Sage Publications.

[bibr71-1471301221997281] Riley R. J. Burgener S. Buckwalter K. C. (2014). Anxiety and stigma in dementia: A threat to aging in place. Nursing Clinics of North America, 49(2), 213-231. doi:10.1016/j.cnur.2014.02.008 24846469 PMC4032087

[bibr72-1471301221997281] Rodgers P. (2018). Co-designing with people living with dementia. CoDesign, 14(3), 188-202. doi:10.1080/15710882.2017.1282527

[bibr73-1471301221997281] Schwartzberg J. G. Fleming M. Oliver C. Vergara K. C. Vangeest J. B. (2005). Evaluating a health literacy kit for physicians. Studies in Communication Sciences, 5(2), 181-189.

[bibr74-1471301221997281] Smith L. Phillipson L. (2020). Thinking through participatory action research with people with late-stage dementia: Research note on mistakes, creative methods and partnerships. International Journal of Social Research Methodology. doi:10.1080/13645579.2020.1810997

[bibr75-1471301221997281] Soong A. Au S. T. Kyaw B. M. Theng Y. L. Tudor Car L. (2020). Information needs and information seeking behaviour of people with dementia and their non-professional caregivers: A scoping review. BMC Geriatrics, 20, 61. doi:10.1186/s12877-020-1454-y 32059648 PMC7023704

[bibr76-1471301221997281] Sørensen L. Waldorff F. Waldemar G. (2008) Early counselling and support for patients with mild Alzheimer's disease and their caregivers: A qualitative study on outcome, Aging & Mental Health, 12(4), 444-450. doi:10.1080/13607860802224342 18791891

[bibr77-1471301221997281] Techwalla (n.d.) How to storyboard your website. https://www.techwalla.com/articles/how-to-storyboard-your-web-site

[bibr78-1471301221997281] Thomas J. E. (1982). Radical adult education: Theory and practice. Nottingham: Department of Continuing Education, University of Nottingham.

[bibr96-1471301221997281] Umapathy H. Bennell K. Dickson C. Dobson F. Fransen M. Jones G. Hunter DJ. (2015). The web-based osteoarthritis management resource my joint pain improves quality of care: A quasi-experimental study. Journal of Medical Internet Research, 17(7): e167. doi:10.2196/jmir.4376 26154022 PMC4526979

[bibr79-1471301221997281] Van Grieken R. Van Tricht M. Koeter M. van den Brink W. Schene A. (2018). The use and helpfulness of self-management strategies for depression: The experiences of patients. Plos One, 13(10), Article e0206262. doi:10.1371/journal.pone.0206262 30359444 PMC6201928

[bibr80-1471301221997281] Vas A. Devi E. Vidyasagar S. Acharya R. Radhakrishna Rau N. George A. Jose T. Nayak B. (2017). Effectiveness of self-management programmes in diabetes management: A systematic review. International Journal of Nursing Practice, 3(5). doi:10.1111/ijn.12571 28758701

[bibr81-1471301221997281] Vernooij-Dassen M. Moniz-Cook E. Woods R. De L. Leuschner A. Zanetti O. Rotrou J. Kenny G. Franco M. Peters V. Iliffe S. (2005). Factors affecting timely recognition and diagnosis of dementia across Europe: From awareness to stigma. Internal Journal of Geriatric Psychiatry, 20(4), 377-386. doi:10.1002/gps.1302 15799080

[bibr82-1471301221997281] Vernooij-Dassen M. Olde Rikkert M. (2004). Personal disease management in dementia care. International Journal of Geriatric Psychiatry, 19(8), 715-717. doi:10.1002/gps.1149 15290693

[bibr83-1471301221997281] Wang G. Marradi C. Albayrak A. van der Cammen T. (2019). Co-designing with people with dementia: A scoping review of involving people with dementia in design research. Maturitas, 127, 55-63. doi:10.1016/j.maturitas.2019.06.003 31351521

[bibr84-1471301221997281] Warner G. Packer T. Kervin E. Sibbald K. Audulv A. (2019). A systematic review examining whether community-based self-management programs for older adults with chronic conditions actively engage participants and teach them patient-oriented self-management strategies. Patient Education and Counselling, 102(12), 2162-2182. doi:10.1016/j.pec.2019.07.002 31301922

[bibr85-1471301221997281] Warner G. Packer T. Villeneuve M. Audulv A. Versnel J. (2015). A systematic review of the effectiveness of stroke self-management programs for improving function and participation outcomes: Self-management programs for stroke survivors. Disability and Rehabilitation, 37(23), 2141-2163. doi:10.3109/09638288.2014.996674 25579669

[bibr86-1471301221997281] Welsby J. Horsfall D. (2011). Everyday practices of exclusion/inclusion: Women who have an intellectual disability speaking for themselves*?* Disability & Society, 26(7), 795–807. doi:10.1080/09687599.2011.618731

[bibr91-1471301221997281] Wiersma E. C. McAiney C. Loiselle L. Hickman K. Harvey D. (2017). Shifting focus: Agency and resilience in a self-management program for people living with dementia. In Clarke C.L. Schwannauer M. Taylor J. Rhynas J. (Eds.) Risk and Resilience: Global Learning across the Age Span. Edinburgh, 33-46.

[bibr87-1471301221997281] World Health Organization . (2015). Ensuring a human right-based approach for people living with dementia. https://www.who.int/mental_health/neurology/dementia/dementia_thematicbrief_human_rights.pdf. doi:10.1177/1524839915571633

